# Nfu1 Mediated ROS Removal Caused by Cd Stress in *Tegillarca granosa*

**DOI:** 10.3389/fphys.2017.01061

**Published:** 2017-12-18

**Authors:** Guang Qian, Yongbo Bao, Chenghua Li, Qingqing Xie, Meng Lu, Zhihua Lin

**Affiliations:** ^1^School of Marine Sciences, Ningbo University, Ningbo, China; ^2^Zhejiang Key Laboratory of Aquatic Germplasm Resources, College of Biological and Environmental Sciences, Zhejiang Wanli University, Ningbo, China

**Keywords:** blood clam, Nfu1, Fe-S clusters, ROS, Cu/ZnSOD, cadmium

## Abstract

The blood clam *Tegillarca granosa*, a eukaryotic bottom-dwelling bivalve species has a strong ability to tolerate and accumulate cadmium. In our previous study, Nfu1 (iron-sulfur cluster scaffold protein), which is involved in Fe-S cluster biogenesis, was shown to be significantly up-regulated under Cd stress, as determined by proteomic analysis. To investigate the function of Nfu1 in cadmium (Cd) detoxification, the function of blood clam Nfu1 (designated as Tg-Nfu1) was investigated by integrated molecular and protein approaches. The full-length cDNA of Tg-Nfu1 is 1167 bp and encodes a protein of 272 amino acid residues. The deduced Tg-Nfu1 protein is 30 kDa contains a conserved Nfu-N domain and a Fe-S cluster binding motif (C-X-X-C). qRT-PCR analysis revealed that Tg-Nfu1 was ubiquitously expressed in all examined tissues; it was up-regulated in the hepatopancreas and gill, and kept a high level from 9 to 24 h after Cd exposure (250 μg/L). Western blot analysis further revealed that the Tg-Nfu1 protein was also highly expressed in the hepatopancreas and gill after 24 h of Cd stress. Further functional analysis showed that the production of ROS was increased and Cu/ZnSOD activity was inhibited in blood clam, treated with the specific Nfu1 siRNA and Cd stress, respectively. These results suggest that Tg-Nfu1 could protect blood clam from oxidative damage caused by Cd stress.

## Introduction

In recent years, with the development of coastal economies, pollution of marine ecosystems by heavy metals has not only caused damage as a result of industrial activity but has seriously limited the sustainable development of aquaculture, particularly on the east coast of China (Wu et al., 2012; Wang et al., 2013). Heavy metals (Cd, Cr, Cu, Hg, and Zn) released into the environment and accumulate in many marine invertebrates, especially in bivalves and gastropods (Abdullah et al., 2007). The pollution of Cadmium (Cd) is increasing serious in the East China Sea due to the drainage of industrial sewage (Bao et al., 2014). The influence of the increased heavy metal content in shellfish on food safety has become a concern with the improvement of health standards.

The marine blood clam (*Tegillarca granosa*) is a bivalve mollusc of the family Arcidae. Because of its delicious taste and medicinal value, it is a major fishery and aquaculture species on the east coast of China and Southeast Asia (Abbas Alkarkhi et al., 2008). As a benthic filter feeder, blood clam has higher Cd accumulation capacity and tolerance to the metal's toxicity than oysters and mussels (Gong et al., 2011), and it is a great object for studying resistance to various natural stresses, such as hypoxia, heavy metals and pollutants (Bao et al., 2014). Numerous studies have reported the toxicological mechanism or enrichment of Cd binding proteins, for example, metallothionein (MT) as bioindicators to response the increase of Cd (Bebianno and Serafim, 1998; Lange et al., 2002) can mobilize and detoxify Cd by forming Cd-thiol complexes (Marasinghe Wadige et al., 2014). Superoxide dismutase (SOD) activity has also been reported as an index to estimate the Cd level (Geret et al., 2002). Recent research found that Vitellogenin (Vg), a protein rich in cys, may play an important role in *T. granosa* metal detoxification (Chen et al., 2017). However, the mechanisms of enrichment and detoxification of Cd in blood clams remain unclear.

As is known to all, ROS production was one of the first steps in Cd-mediated cytotoxicity, which causes a series of biological reactions (Cuypers et al., 2010). In pacific oyster, antioxidant enzymes play important roles in the physiological changes related to metabolism and cell protection when exposed to Cd (Jo et al., 2008). Additionally, SOD plays a key role in protecting aerobic organisms against oxidative damage (Strain et al., 1998). Iron-sulfur proteins are particularly oxygen sensitive, and their inorganic cofactors frequently undergo ROS-induced decomposition reactions (Bruska et al., 2015). Nfu1, an iron-sulfur (Fe-S) cluster protein, is necessary for lipoic acid biosynthesis and respiratory chain complex activities (Ferrer-Cortès et al., 2013). Transcriptional activators Yap1 and Yap2 (Yes associated protein) can induce a battery of antioxidant genes expression under oxidative stress, including thioredoxin, thioredoxin reductase, and glutathione reductase (Fernandes et al., 1997), and as high-copy suppressors of Nfu1 mutant cells, they act a functional role for Nfu1 during oxidative stress (Melber et al., 2016). Additionally, Fe-S cluster synthesis may be related to sulfur metabolism because of its ability to transport sulfide (Liu et al., 2009), and sulfur metabolism has been reported to be related to Cd detoxification (Clemens, 2006).

In our previous work, we found that Nfu1 protein expression was significantly increased in blood clam under Cd stress, as measured by iTRAQ proteome analysis (Bao et al., 2016). To investigate whether Tg-Nfu1 plays a role in Cd resistance, the full-length cDNA of Tg-Nfu1 was cloned and analyzed, its expression pattern after Cd stress was also evaluated, as ROS production and Cu/ZnSOD activity after exposure to Cd stress and Tg-Nfu1 interference. This study could provide cognition to well understand the mechanism of heavy metal detoxification in blood clam.

## Materials and methods

### Experimental animals and challenge experiment

Blood clams, averaging approximately 30 mm in shell length, were collected from a bivalve farm in Ningbo, for challenge experiment and Nfu1 silencing experiment. Collected clams were acclimatized in tank for seven days with 25°C temperature and 30‰ salinity of seawater. The seawater added with chaetoceros stock was changed daily. For the Cd challenge experiment, blood clams were divided into two tanks, and one was exposed to Cd with a final gradient concentration of 250 μg/L which followed our previous study (Bao et al., 2016). The other tank contained the control group treated with seawater. After 0, 3, 6, 9, 12, and 24 h of Cd exposure, three clams were pooled together as one replicate, total three replicates, and six tissues of blood clam including hepatopancreas, gill, foot, adductor muscle, mantle and hemocytes were collected in liquid nitrogen, and then store at −80°C until further analysis. Cd solution was disposed by adding EDTA after experience. All animals here are commercially cultured, and all the experiments were conducted in accordance with the recommendations in the Guide for the Care and Use of Laboratory Animals of the National Institutes of Health. The study protocol was approved by the Experimental Animal Ethics Committee of Ningbo University, China.

### Nfu1 silencing

A small interfering RNA (siRNA) targeting Nfu1 was designed (Table 1) and synthesized by GenePharma (Shanghai, China), and SiRNA were diluted in 100 μl of saline solution with a concentration of 0.5 μg/μl. For the Nfu1 silencing experiment, rest of acclimatized clams were anesthetized in a MgCl2 solution (3/5 fresh water, 2/5 seawater and 50 g/L MgCl2) for 5 h and divided into two tanks. Anesthetized blood clams from one tank were injected in the visceral mass (digestive gland surrounded by mantle-gonad tissue) with 100 μl of transfection solution (Fabioux et al., 2009). The other tank's clams contained the control were injected with 100 μl of saline solution without siRNA. Healthy blood clams were maintained in tanks after injection, and after 24 h, haemocytes of all blood clams were sampled from two tanks, and each ten clams in every tank were collected for the analysis of Nfu1 expression, ROS production and Cu/ZnSOD activity, respectively. Every clam presented a replicate, total ten replicates.

**Table 1:** Primers and interference sequence information in the present study.

**Primer**	**Sequence (5′–3′)**	**Used for**
Tg-Nfu1 3-1	CAACAGTTCAGGAGGATGGTGGA	3′RACE
Tg-Nfu1 3-2	GCACAGTCATCACATGCCTCACC	
Tg-Nfu1 5-1	CACCATCTATCCGAAACAACTGC	5′RACE
Tg-Nfu1 5-2	TCTCCACCATCCTCCTGAACTGT	
Tg-Nfu1 F	GGCTGTGTCTGATGGAGGTT	Real-time PCR
Tg-Nfu1 R	CAGTCATCACATGCCTCACC	
Tg-18sRNA	CTTTCAAATGTCTGCCCTATCAACT	Real-time PCR
Tg-18sRNA	TCCCGTATTGTTATTTTTCGTCACT	
**siRNA INTERFENENCE SEQUENCE**		
Tg-Nfu1 siRNA	UUAACUUAACAAUUCCAUCUU	Tg-Nfu1 interference
	GAUGGAAUUGUUAAGUUAAAG	

### Cloning of full-length cDNA of Nfu1

The tissues sampled in Cd stress and the haemocytes sampled in Nfu1 silencing were collected for RNA extraction using the RNAiso Plus Kit (TaKaRa, Japan). The quality and quantity of each RNA were determined by NanoVue Plus (GE, USA), and the A260/A280 ratio of RNA at 1.8–2.0 were used for cDNA synthesis using the PrimeScript™ RT regent Kit (TaKaRa, Japan) according to the protocol (1 μg total RNA per 20 reaction). The partial cDNA sequence of Nfu1 was extracted from our previous blood clam transcriptome data (Bao et al., 2016), and gene specific primers (Table 1) were designed for a 5′ and 3′ RACE experiment. PCR products were ligated into the pMD18-T vector (TaKaRa, Japan). Competent cells *Escherichia coli* DH5α which were transferred with recombinant vector were used for positive clones screening by PCR analysis and the clones were bi-directionally sequenced.

### Sequence analysis of the Nfu1 cDNA

The Nfu1 cDNA sequences were analyzed using the Conserved Domain Database and BLAST algorithm at the NCBI (http://www.ncbi.nlm.nih.gov/blast), and the potential N-glycosylation sites were predicted by the NetNGlyc 1.0 server (http://www.cbs.dtu.dk/services/NetNGlyc/). The open reading frame of Tg-Nfu1 was found using the DNAMAN 6.0 program, and also analyzed its amino acid sequence. Multiple alignments of each protein were performed using the Mega 7.0 program. The domains of the Tg-Nfu1 amino acid sequence were detected using the simple modular architecture research tool (SMART) program (http://smart.embl-heidelberg.de/).

### Real-time quantitative PCR

The Tg-Nfu1 expression analysis of tissue distribution, time-course and Tg-Nfu1 silencing was conducted by qRT-PCR experiment using SYBR Green detection chemistry (TaKaRa, USA) performed in ABI 7500 Fast (Thermo Fisher Scientific, USA). The primer information for the qRT-PCR is shown in Table 1. 18SrRNA served as an internal control to normalize the Nfu1 gene for quantification. PCR reactions were carried out in a total volume of 20 μl containing 2 μl of cDNA, 2 μl of each primer (10 mM), 6 μl of RNase-free water, and 10 μl of the SYBR Green PCR Master Mix (TaKaRa, Japan). The cycling conditions were 94°C for 5 min, followed by 40 cycles of 94°C for 15 s and 60°C for 40 s. At the end of the PCR cycles, melting curve analyses were performed. The Ct value is defined as the fractional cycle number at which the fluorescence passes the fixed threshold. Each sample was analyzed in 3 triplicates.

### Assay of respiratory burst

Intracellular ROS production was measured using spectrophotometric method (Chen et al., 2005). Five-hundred microliters of haemocytes cells was mixed with nitro blue tetrazolium (Sangon, China) and phorbol 12-myristate 13-acetate (Sangon, China) with a final concentration of 0.1% and 0.01 mM, respectively. The sample was incubated at room temperature for 1 h, the supernatant was removed by centrifugation at 540 × g for 10 min, and cells were fixed with 35% methanol and washed twice with 70% methanol. Finally, the cells were re-suspended in 1.3 mL of solution containing 0.92 M potassium hydroxide and 54% dimethyl sulfoxide. The optical density was determined at 625 nm to represent the ROS level in each sample.

### Assay of Cu/Zn SOD activity

Five hundred microliter of haemocytes in each replicate was centrifuged at 3,000 rpm for 10 min. The supernatant collected for the quantification by Bradford method (Kruger, 1994) was used for enzymatic assay, and Cu/ZnSOD activity was detected with a Cu/ZnSOD assay kit (Jiancheng, China) according to the protocol. The sample that produced 45–50% inhibition was defined as one unit (U) of SOD for Cu/ZnSOD activity assay.

### Western blot analysis

A Tg-Nfu1 polyclonal antibody was prepared against our recombinant Tg-Nfu1, the domain sequence of Tg-Nfu1 was cloned to pET-28a vector, and the recombinant vector was transferred in DH5α (TaKaRa, Japan) for amplification, and then transferred in BL21 DE3 (TaKaRa, Japan) for protein expression. The expressed protein was purified by Ni NTA column using Ni-NTA Sefinose™ Resin (BBI, USA), and the purified protein was sent to company (Huabio, China) for antibody preparation. The antiserum was harvested from rabbit and stored at −20°C for further experiments.

For the Western blot assay, total protein of blood clam's hepatopancreas and gill was extracted from the clams receiving 24 h of Cd stress or the control culturing 24 h in seawater by using Total Protein Extraction Kit (Sangon, China) according to the protocol. The protein concentration was quantified by the BCA Protein Assay Kit (Sangon, China). Fifty microgram of protein was subjected to 12% SDS-polyacrylamide gel for electrophoresis, then transferred onto a 0.45 mm PVDF membrane for 3 h. After blocking with 5% skimmed milk in TBST (50 mmol/L Tris-HCl, 150 mmol/L NaCl, and 20% Tween-20) at 4°C overnight, the membrane was incubated with Tg-Nfu1 or β-actin antibodies diluted at 1:400 in 5% bovine serum albumin (BSA) at room temperature for 2 h, then were washed three times with TBST and incubated with HRP-labeled anti-rabbit lgG (1:3,000) in 5% BSA for 1.5 h at room temperature. The membrance were washed three times with TBST for 10 min each, and were incubated in Western Lighting - ECL substrate (Advansta, China) prior to exposure to X-OMAT AR X-ray film (Eastman Kodak, Rochester, NY). The results were derived from statistical analysis of three independent experiments, and the statistics of Western Blot analyzed by Image Pro Plus program 6.0 according to the size of stripe.

### Statistical analysis

The mRNA expression levels of the Tg-Nfu1 were calculated by the 2^−ΔΔ*CT*^ method, n-fold change relative to corresponding control were represents the expression value. The statistical significance differences between challenged and control groups were determined using one-way analysis of variance (ANOVA) followed with multiple Duncan tests and the data were showed as the mean ± S.D. Any significant differences are indicated with an asterisk at *P* < 0.05 and two asterisks at *P* < 0.01.

## Results

### Analysis of the Tg-Nfu1 gene sequence

**Figure 1:**
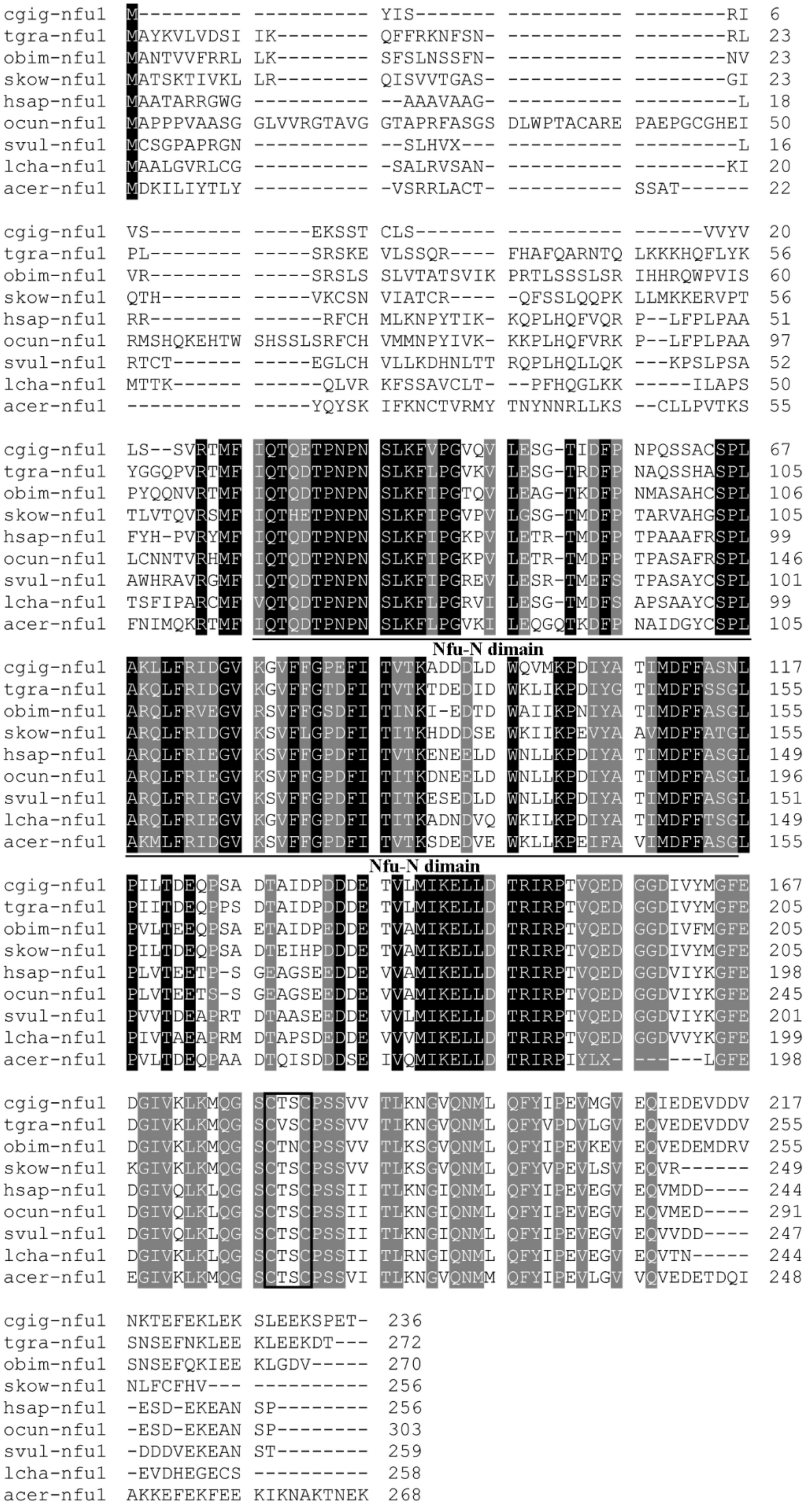
Multiple sequence alignment of Nfu1 from *Tegillarca granosa* (Tg-Nfu1) with other known Nfu1 amino acid sequences. *Crassostrea gigas* (Cgig, XP_011432047.1), *Octopus bimaculoides* (Obim, XP_014784246.1), *Saccoglossus kowalevskii* (Skow, XP_006814052.1), *Homo sapiens* (Hsap, NP_001002755.1), *Oryctolagus cuniculus* (Ocun, XP_014784246.1), *Sturnus vulgaris* (Svul, XP_014746903.1), *Latimeria chalumnae* (Lcha, XP_005988282.1), *Apis cerana* (Acer, XP_016914363.1). Identities are shaded dark and similarities are shaded gray. The Nfu-N domain regions are underlined and the Fe-S cluster binding motif (C-X-X-C) is boxed.

The 3′- and 5′- ends of Nfu1 were cloned using the SMART-RACE approach. Full-length Tg-Nfu1 cDNA was 1,167 bp with a 5′ untranslated region (UTR) of 26 bp, an open reading frame (ORF) of 819 bp encoding 272 amino acids (Figure S1), and a 3′ UTR of 322 nucleotides with a possible polyadenylation signal (AATAAA) 13 bp upstream of the polyadenylation tail (Figure S1). The sequence was deposited in GenBank under accession number MG596304. The calculated molecular mass of the deduced mature Nfu1 was 30 KDa, with a theoretical pI of 4.75. The amino acid sequence of Tg-Nfu1 was aligned with selected Nfu1 sequences from the NCBI database using the ClustalW method. Amino acid sequence analysis showed that Tg-Nfu1 possesses a potential N-glycosylation site close to the N-terminus, a characteristic feature of Nfu-N domains, which is found in the region including residues 67–154 in other reported Nfu1s. Tg-Nfu1 was also found to contain a Fe-S cluster binding motif (C-X-X-C) at position 217 of the protein, which is highly conserved among all species (Figure 1). BLAST analysis revealed that Tg-Nfu1 shared approximately 72–83% overall identity with its homologs from vertebrates and invertebrates, for example, 72% with *Homo sapiens* and 83% with *Crassostrea gigas*.

**Figure 2:**
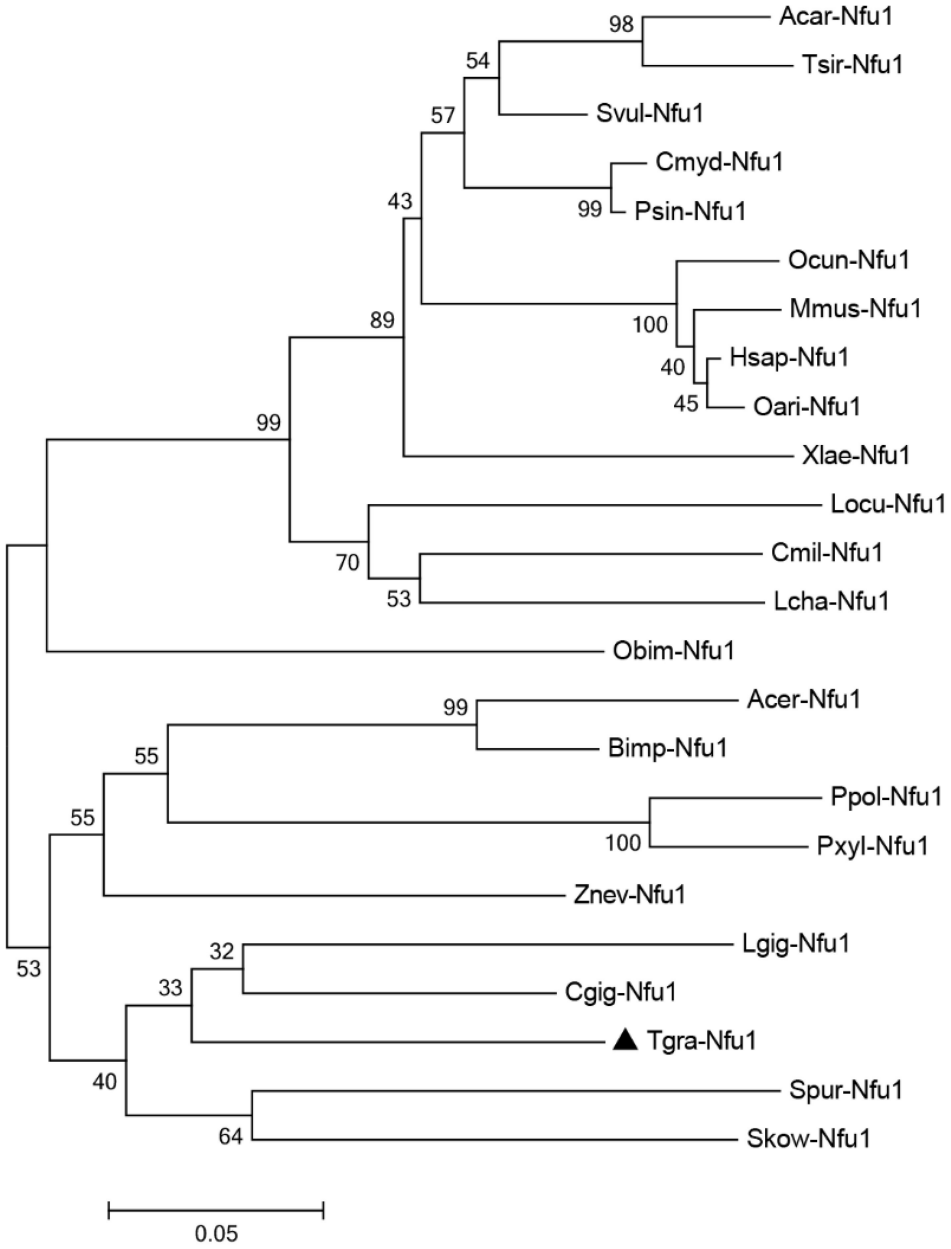
Neighbour-joining phylogenetic analysis of the Nfu1 amino acid sequence from different species. The abbreviations and GenBank accession numbers used to construct the phylogenetic tree are given in Table S1. One thousand bootstrap trials were run using the neighbor-joining algorithm (Mega 7 program).

To determine the phylogenetic relationship of Tg-Nfu1 with other counterparts, a phylogenetic tree was constructed by the neighbor-joining (NJ) algorithm, as shown in Figure 2. The Nfu1 subgroup is divided into two major branches: vertebrates and invertebrates. Tg-Nfu1 was first clustered with Nfu1 from *C. gigas* and then formed a sister subgroup with arthropods. Tg-Nfu1 was somewhat close to homologs from invertebrates, such as echinodermata (*Strongylocentrotus purpuratus*) and insects (*Apis cerana*), but was distant from homologs from fish, aves and mammals.

### Tissue expression profiles after Cd challenge of Tg-Nfu1

**Figure 3:**
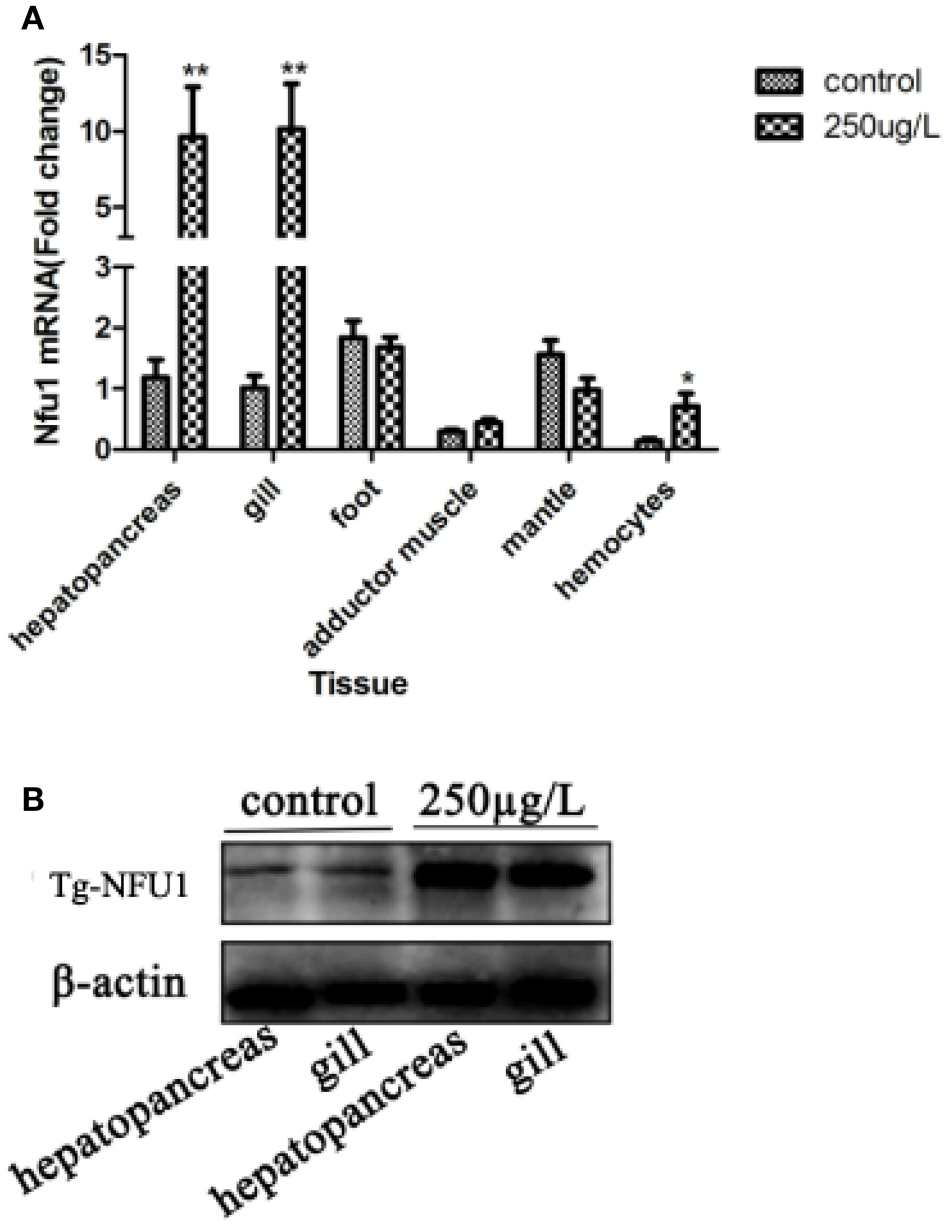
Tg-Nfu1 gene and Tg-NFU1 protein expression after 24 h of Cd stress. **(A)** Real-time analysis of the amount of Tg-Nfu1 transcript relative to 18rRNA transcript in various tissues. Expression levels were calibrated against gill (control). Vertical bars represent the means ± S.D., *n* = 3. Asterisks indicate significant differences: ^**^p < 0.01; **(B)** Tg-NFU1 protein expression in hepatopancreas and gill. Control: treated with seawater for 24 h; 250:250 μg/L of Cd stress for 24 h.

The Tg-Nfu1 tissue distribution is shown in Figure 3A. The Tg-Nfu1 mRNA transcript was detected in all examined tissues, including the hepatopancreas, gill, foot, adductor muscle, mantle, and haemocytes. In the treatment group, Tg-Nfu1 mRNA expression was significantly upregulated in the hepatopancreas and gill after Cd stress (*P* < 0.01), and there was also an obvious upregulation in haemocytes (*P* < 0.05). Besides, we did not detect obvious changes in the foot and mantle. Prokaryotic expression and purification of recombinant Tg-Nfu1 protein was showed in Figure S2, the molecular weight of recombinant protein that contained Tg-Nfu1domain was about 16 KDa, according to protein electropherogram. Western blot analysis revealed that Tg-NFU1 protein was also present in the hepatopancreas and gill and showed 2.9- and 1.3-fold increases after 24 h of Cd stress (Figure 3B).

### Temporal expression analysis of Tg-Nfu1 under Cd stress

**Figure 4:**
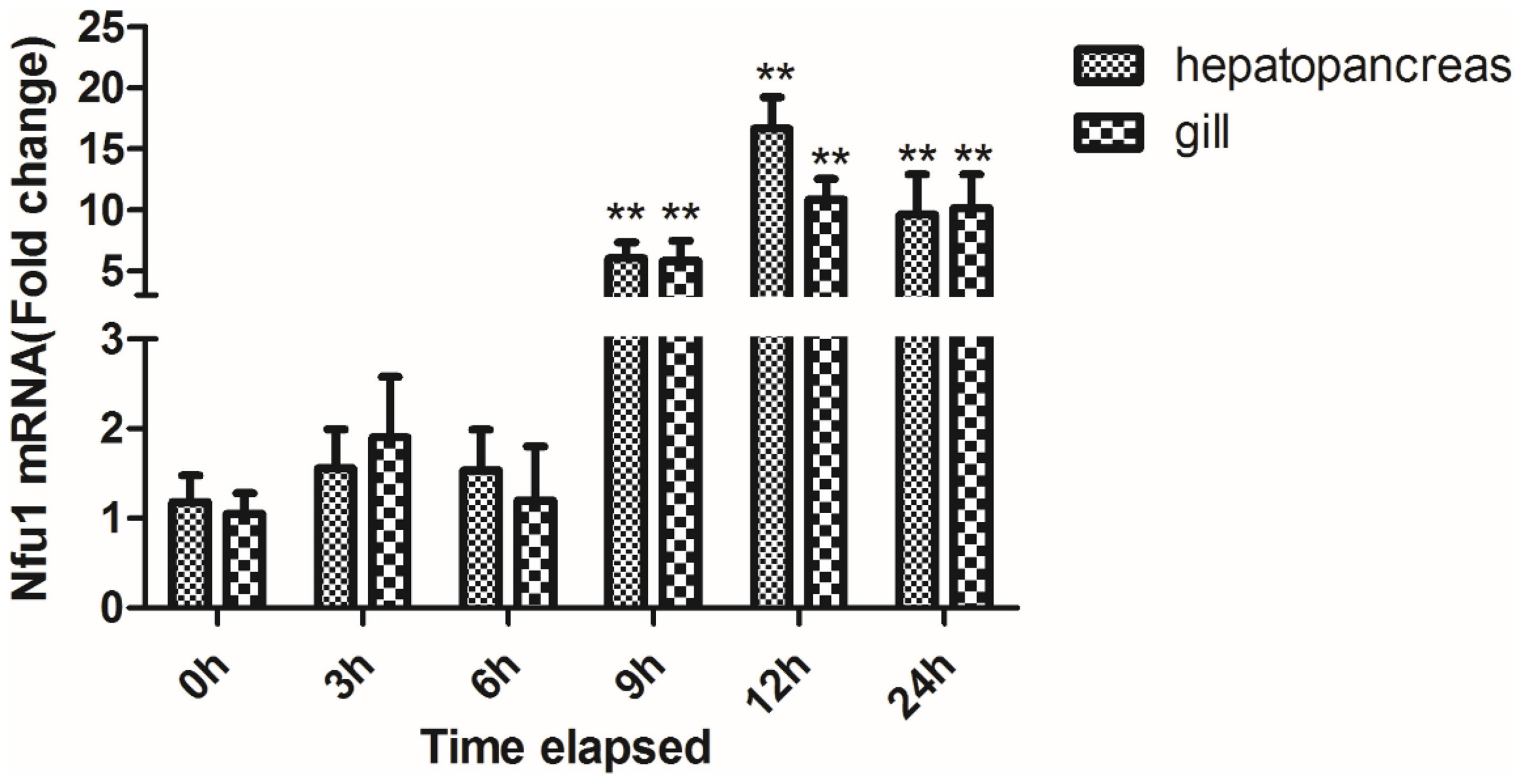
Time-course expression of the Tg-Nfu1 gene in the hepatopancreas and gill under Cd stress. Values are given as the mean ± S.D., *n* = 3. Asterisks indicate significant differences: ^**^p < 0.01.

Temporal expression of the Tg-Nfu1 transcripts in the hepatopancreas and gill under Cd stress is shown in Figure 4. The expression level of Tg-Nfu1 mRNA did not change in the first 6 h, but then upregulated obviously in the hepatopancreas and gill at 9 h (*P* < 0.01). Tg-Nfu1 reached peak expression at 12 h (*P* < 0.01) and maintained a high expression level until 24 h.

### Cu/ZnSOD activity and ROS production toward Cd stress

**Figure 5:**
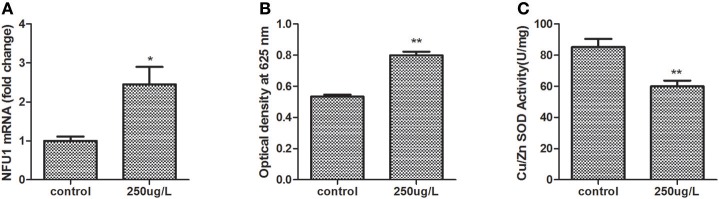
Assay of ROS production and Cu/ZnSOD activity in blood clam's hemocytes under Cd stress after 24 h. Control, Cd stress control; 250 μg/L, Cd stress with a concentration of 250 μg/L. Asterisks indicate significant difference: ^*^p < 0.05, ^**^p < 0.01. **(A)** Tg-Nfu1 expression; **(B)** ROS production; **(C)** Cu/ZnSOD.

Oxidative stress is a long-lasting effect of heavy metal stress, and Cu/ZnSOD plays a critical role in protecting aerobic organisms against oxidative damage. Therefore, the respiratory burst and Cu/ZnSOD were investigated after Cd stress (Figure 5). The Tg-Nfu1 expression level was up-regulated compared with the control group (Figure 5A), and the intracellular ROS level was also significantly increased to 33.1% (Figure 5B), and the level of Cu/ZnSOD activity was decreased to 29.5% (Figure 5C).

### Tg-Nfu1 increases Cu/ZnSOD activity by decreasing ROS

**Figure 6:**
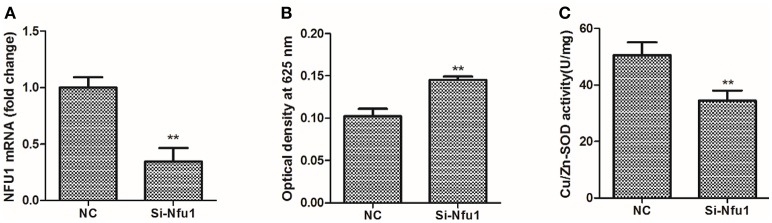
Cu/ZnSOD activity and ROS production in blood clam's hemocytes after Tg-Nfu1 silencing. Si-Nfu1, Tg-Nfu1 silencing; NC, Tg-Nfu1 silencing control. Asterisks indicate significant differences: ^**^p < 0.01. **(A)** Tg-Nfu1 expression; **(B)** ROS production; **(C)** Cu/ZnSOD.

ROS production after Tg-Nfu1 silencing in blood clams is shown in Figure 6. The respiratory burst was elevated to 29.5% (Figure 6B); by contrast, Cu/ZnSOD was decreased by 31.7% (Figure 6C) after Tg-Nfu1 knock-down (Figure 6A).

## Discussion

Molluscs have the ability to accumulate, concentrate, and integrate heavy metals and used as indicators of ocean health in worldwide, (Bebianno et al., 1993). Blood Clam as a benthic filter feeder has a higher Cd accumulation capacity and tolerance to metal toxicity than other shellfish including mussels and oysters (Bao et al., 2014). However, the mechanisms underlying these phenomena are not yet well understood. Bao et al. (2016) found that sulfur-related metabolism genes, such as sulfotransferase (Sult1b1) and protein disulfide-isomerase, were significantly up-regulated after Cd challenge using iTRAQ proteome analysis in blood clams, suggesting that sulfur-related metabolism might also be involved in the Cd-induced response and detoxification of Cd in animals. Our study including identification of N-terminal glycosylation sites (Figure 1), a CxxC motif and Nfu-N domain as known to be a [4Fe-4S] cluster at a homodimer interface (Tong et al., 2003; Angelini et al., 2008; Bandyopadhyay et al., 2008; Gao et al., 2013). Cys-217 and Cys-220 residues (CxxC) from the Nfu-N domain were expected to be functionally important as they are strictly conserved in all members of Nfu family, and such a motif is essential for the activity of most protein of the thioredoxin superfamily and some zinc finger proteins (Iuchi and Kuldell, 2005; Pan and Bardwell, 2006). All of these results were also found in Tg-Nfu1, indicating that the newly identified Tg-Nfu1 is similar to other organisms.

Real-time PCR analysis demonstrated that Tg-Nfu1 mRNA was expressed in all of the studied tissues, and quickly increased after 9 h of Cd stress; western blot analysis also showed an increase for the level of protein after 24 h of Cd stress. As a nonessential metal, Cd cannot be degraded or biotransformed; therefore, it easily accumulates in upper trophic organisms (Henson and Anderson, 2000; Nasreddine and Parent-Massin, 2002). This differential Cd accumulation of tissues has also been identified in fish (Dobaradaran et al., 2013), crustacea (Silvestre et al., 2005), and mammals (Habeebu et al., 1998). Gills are the main channel for the enrichment of metal ions from aquatic organisms and are the main organ for enriching heavy metals in the short-term (Paul-Pont et al., 2010). In *Mytilus galloprovincialis*, heavy metals can cause breakage and fusion of gill silk, which affects the transport of heavy metals to other organs (Domouhtsidou and Dimitriadis, 2000). Thus, the hepatopancreas and gill are important for the modulation and clearance of cadmium (Haberkorn et al., 2010). Here, the high expression of Tg-Nfu1 in hepatopancreas and gill under Cd stress indicted that Tg-Nfu1 might be also associated with the response to Cd. Additionally, the universal expression of Tg-Nfu1 suggests that it is might involve in various metabolic pathways.

In this study, ROS production and Cu/ZnSOD activity were detected in blood clams after 24 h of Cd stress. Oxidative stress can disrupt cellular macromolecules by disturbing cellular redox balance, and leading to necrosis (Auclair and Gagné, 2014). The increase in blood clam ROS levels after Cd stress showed that Cd could induce oxidative stress and cause damage to the body. The effects of cadmium-induced oxidative stress in animals, plants, cells and tissues are outlined in several reviews (Prasad and Hagemeyer, 1999; Waisberg et al., 2003; Bertin and Averbeck, 2006; Joseph, 2009). Funes et al. (2005) reported that mollusks (oysters *Crassostrea angulata* and mussels *M. galloprovincials*) from areas that had severe heavy metal contamination had a higher activation in CAT, SOD, and GPX. Other studies reported that Cd could induce antioxidant gene in fish, such as brown trout, *Salmo trutta* (Hansen et al., 2007), and sea bass, *Dicentrarchus labrax* (Roméo et al., 2000).

It is generally agreed that oxidative stress plays an important role in acute Cd poisoning (Pathak and Khandelwal, 2006), and SOD are the first and most important line of defense against ROS, especially superoxide anion radicals (Zelko et al., 2002). SOD activity during Cd exposure has been studied intensively and both increases as well as decreases are described in literature (Jurczuk et al., 2004; Yalin et al., 2006; Rocha et al., 2015). These apparent discrepancies can be attributed to different exposure conditions as well as the organ system studied, leading to a different outcome (Cuypers et al., 2010). In this study, the activity of Cu/ZnSOD was inhibited because of the replacement of zinc with Cd in Zn/Cu superoxide dismutase after Cd-induced stress (Kusumoto et al., 1991; Casalino et al., 1997; Riordan et al., 2005), and the resulting ROS accumulated in the organism.

Fe-S clusters are essential for many cellular processes, including aerobic respiration, metabolite biosynthesis, ribosome assembly and DNA repair, and their inorganic cofactors frequently undergo ROS-induced decomposition reactions (Bruska et al., 2015). For most [4Fe-4S] client proteins, Nfu1, an alternate scaffold protein (Cameron et al., 2011), appears to have evolved to shield its [4Fe-4S] cluster from endogenous oxidants during the cluster transfer step (Melber et al., 2016). In this study, we reduced expression of Tg-Nfu1 using RNAi technology and detected the activity of Cu/ZnSOD as well as production of ROS. Production of ROS increased after Nfu1 interference in blood clams, which indicated that Tg-Nfu1 could decrease the production of ROS and prevent oxidative damage to [4Fe-4S] (Melber et al., 2016). Additionally, Nfu1 has been implicated in lipolic acid biosynthesis and respiratory chain complex activity (Navari-Izzo et al., 2002). Lipolic acid interacts with other metal-binding proteins and not only functions in the detoxification of Cd and other heavy mental but also plays an important role in antioxidant activity (Liu et al., 2009).

There is a correlation between sulfur metabolism and cadmium. Many studies have shown that Cd induces sulfate uptake and that sulfur compounds are involved in the detoxification of Cd in plants (Clemens, 2006). the synthesis of phytochelatins (PCs) can be rapidly induces under Cd exposure in higher plants, which is synthesized using glutathione (GSH) (Steffens, 1990). In animals, GSH, as a metal-binding protein, plays an essential role in the detoxification of Cd and many other metals (Rauser, 1995), and its functions as an important thiol, which may be involved in sulfur metabolism and can mobilize and detoxify Cd by forming Cd-thiol complexes inside the cell (Zalups and Ahmad, 2003). Nfu1, as a persulfide reductase, is associated with sulfide transfer (Tsai and Barondeau, 2010), which is provided by Nfs1 cysteine along with its effector proteins during Fe-S cluster synthesis (Nussbaum et al., 1988; Gerber et al., 2003; Biederbick et al., 2006; Lill and Mühlenhoff, 2006). In this study, the high expression of the Nfu1 gene indicated that cadmium might promote the synthesis of Fe-S clusters by promoting sulfur compounds, thereby maintaining normal cellular metabolism in the blood clam, and this mechanism needs further research.

In conclusion, Nfu1 from the benthic bivalve blood clam was characterized at the sequence level for the first time in the present study. In blood clams, Cd induces both damaging and repair processes in which the cellular redox status plays a crucial role in the cellular (Cuypers et al., 2010). On the one hand, cadmium stress could reduce Cu/ZnSOD activity and cause oxidative damage by producing more reactive oxygen species (Kusumoto et al., 1991; Casalino et al., 1997; Riordan et al., 2005), on the other hand, Cd also induced repair process which clams expressed more Nfu1 to against or eliminate ROS (Wang et al., 2016). The high expression of Tg-Nfu1 after Cd stress suggests that could protect blood clam from oxidative damage caused by Cd stress. Besides, sulfur-related metabolism might also be involved in the Cd-induced response and detoxification of Cd in animals. More studies are necessary to elucidate the roles and related mechanisms of Nfu1 in metal detoxification.

## Author contributions

YB and ZL conceived and designed the experiments. QX and GQ analyzed the data. GQ, CL., and ML performed the experiments and contributed reagents/materials/analysis tools. YB and GQ wrote the paper. All authors reviewed the manuscript.

### Conflict of interest statement

The authors declare that the research was conducted in the absence of any commercial or financial relationships that could be construed as a potential conflict of interest.

## Supplementary material

Figure S1 The nucleotide and deduced amino acid sequence of Tg-Nfu1. The nucleotides and amino acids are numbered from the first base or residues, respectively. The start and stop codons and polyadenylation signal are boxed, the N-glycosylation is shaded with gray, and the domain of Tg-Nfu1 is underlined.

Figure S2 Prokaryotic expression and purification of recombinant Tg-NFU1 protein. **(A)** Tg-NFU1 protein prokaryotic expression. 1: mark; 2: control; 3–7: induce with 3, 4, 5, 6, 12 h; 8: supernatant; 9: inclusion body. **(B)** Tg-NFU1 purification. 1: mark; 2: supernatant; 3: effluent; 4-6: washing buffer; 7-9: elution buffer.

Table S1 Species and GenBank accession numbers of NFU1 sequence used for phylogenetic analysis.
