# Supplementary figures and images for: Nfu1 Mediated ROS Removal Caused by Cd Stress in Tegillarca granosa
Source: Front Physiol. 2017 Dec 18;8:1061. doi: 10.3389/fphys.2017.01061 (PMC5741617; doi:10.3389/fphys.2017.01061)

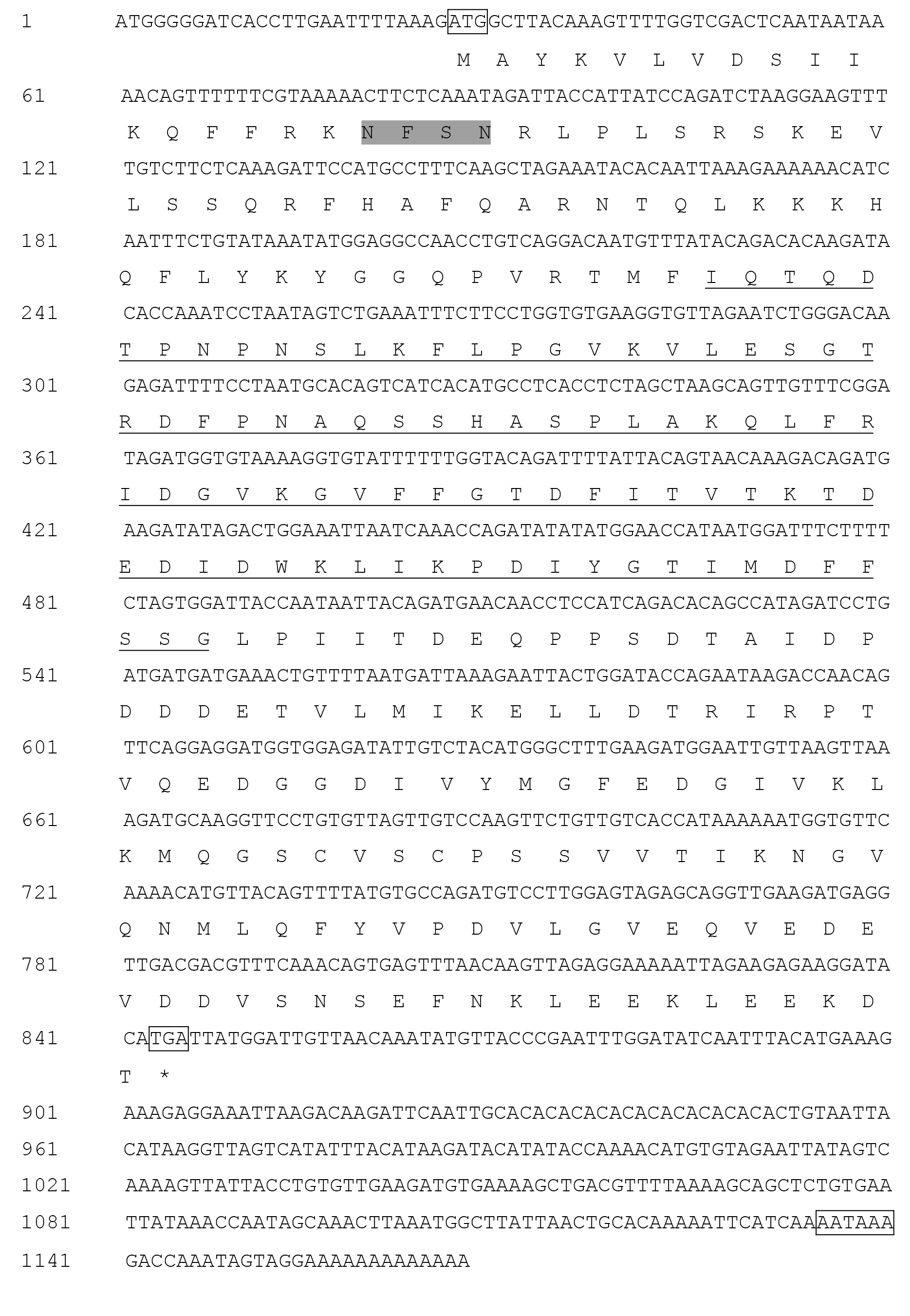

Supplement: Figure S1 — The nucleotide and deduced amino acid sequence of Tg-Nfu1. The nucleotides and amino acids are numbered from the first base or residues, respectively. The start and stop codons and polyadenylation signal are boxed, the N-glycosylation is shaded with gray, and the domain of Tg-Nfu1 is underlined. [file Image1.tif]

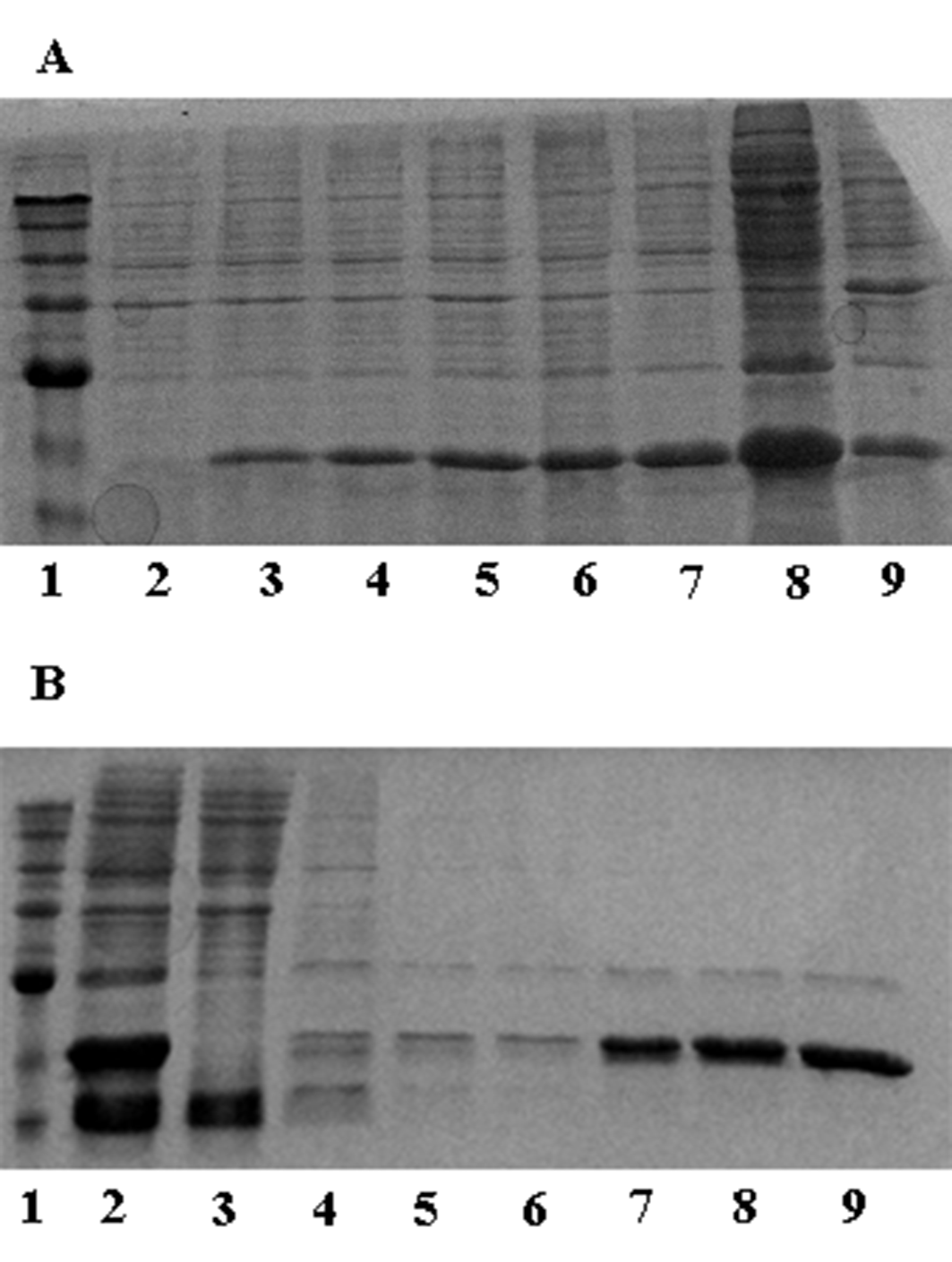

Supplement: Figure S2 — Prokaryotic expression and purification of recombinant Tg-NFU1 protein. (A) Tg-NFU1 protein prokaryotic expression. 1: mark; 2: control; 3–7: induce with 3, 4, 5, 6, 12 h; 8: supernatant; 9: inclusion body. (B) Tg-NFU1 purification. 1: mark; 2: supernatant; 3: effluent; 4-6: washing buffer; 7-9: elution buffer. [file Image2.TIF]
