# Supplementary material for: Nfu1 Mediated ROS Removal Caused by Cd Stress in Tegillarca granosa
Source: Front Physiol. 2017 Dec 18;8:1061. doi: 10.3389/fphys.2017.01061 (PMC5741617; doi:10.3389/fphys.2017.01061)
Supplement: Table S1 — Species and GenBank accession numbers of NFU1 sequence used for phylogenetic analysis. [file Table1.DOCX]

**Table S1** Species and GenBank accession numbers of NFU1 sequence used for phylogenetic analysis.

| Species | GenBank no | Abbreviation-type | Size |
| --- | --- | --- | --- |
| *Anolis carolinensis* | XP_003229651.2 | Acar | 257 |
| *Apis cerana* | XP_016914363.1 | Acer | 268 |
| *Bombus impatiens* | XP_003485658.1 | Bimp | 275 |
| *Callorhinchus milii* | XP_007909171.1 | Cmil | 241 |
| *Chelonia mydas* | XP_007065535.1 | Cmyd | 233 |
| *Crassostrea gigas* | XP_011432047.1 | Cgig | 236 |
| *Homo sapiens* | NP_001002755.1 | Hsap | 254 |
| *Latimeria chalumnae* | XP_005988282.1 | Lcha | 253 |
| *Lepisosteus oculatus* | XP_015193984.1 | Locu | 256 |
| *Limulus polyphemus* | XP_013781654.1 | Lpol | 222 |
| *Lottia gigantea* | XP_009045271.1 | Lgig | 265 |
| *Mus musculus* | NP_064429.2 | Mmus | 255 |
| *Octopus bimaculoides* | XP_014784246.1 | Obim | 273 |
| *Oryctolagus cuniculus* | XP_017196194.1 | Ocun | 301 |
| *Ovis aries* | XP_014949363.1 | Oari | 297 |
| *Papilio polytes* | XP_013140838.1 | Ppol | 267 |
| *Pelodiscus sinensis* | XP_006119161.1 | Psin | 252 |
| *Plutella xylostella* | XP_011549193.1 | Pxyl | 279 |
| *Saccoglossus kowalevskii* | XP_006814052.1 | Skow | 256 |
| *Strongylocentrotus purpuratus* | XP_011679926.1 | Spur | 209 |
| *Sturnus vulgaris* | XP_014746903.1 | Svul | 258 |
| *Thamnophis sirtalis* | XP_013908736.1 | Tsir | 201 |
| *Xenopus laevis* | XP_018110658.1 | Xlae | 264 |
| *Zootermopsis nevadensis* | KDR18183.1 | Znev | 277 |
